# Understanding hepatitis B, hepatitis C and HIV among people who inject drugs in South Africa: findings from a three-city cross-sectional survey

**DOI:** 10.1186/s12954-019-0298-2

**Published:** 2019-04-11

**Authors:** Andrew Scheibe, Katherine Young, Lorraine Moses, Rudolph L. Basson, Anna Versfeld, C. Wendy Spearman, Mark W. Sonderup, Nishi Prabdial-Sing, Jack Manamela, Adrian J. Puren, Kevin Rebe, Harry Hausler

**Affiliations:** 1 TB HIV Care, 11 Adderley Street, Cape Town, South Africa; 2 IQVIA South Africa, CX Building 1021 Lenchen Ave North, Centurion, Pretoria, South Africa; 3 Division of Hepatology, Department of Medicine, Faculty of Health Sciences, University of Cape Town, Old Main Building, Groote Schuur Hospital, Observatory, Cape Town, South Africa; 4 National Institute for Communicable Diseases, 1 Modderfontein Road, Sandringham, Johannesburg, South Africa; 5 Division of Virology, School of Pathology, University of the Witwatersrand, Johannesburg, South Africa; 6 Anova Health Institute, 12 Sherborne Road, Parktown, Johannesburg, South Africa

**Keywords:** Hepatitis C, Hepatitis B, HIV, HBV, HCV, People who inject drugs, Key populations, South Africa

## Abstract

**Background:**

People who inject drugs (PWID) are at high risk for hepatitis C (HCV), hepatitis B (HBV) and HIV without accessible harm reduction programmes. Coverage of needle and syringe and opioid substitution therapy (OST) services in South Africa is below global recommendations and no hepatitis services exist for PWID. We assessed HCV, HBV and HIV prevalence and risk factors among PWID accessing harm reduction services in Cape Town, Durban and Pretoria to inform policy and programming.

**Methods:**

We conducted a cross-sectional survey among PWID in these cities between August 2016 and October 2017. Participants were opportunistically sampled while accessing services. Study team members administered a questionnaire that assessed sociodemographic characteristics, drug use and sexual risk practices. We tested for HCV (antibody, viral load and genotype), HBV surface antigen (HBsAg) and HIV. Bivariate and multivariate analyses assessed associations with HCV serostatus.

**Results:**

Nine hundred and forty-three PWID were included in the per protocol analysis. The majority (87%, 819/943) were male, the overall median age was 29 and most lived on the street (66%, 626/943). At last injection, 77% (722/943) reported using a new needle and syringe and 17% (163/943) shared equipment. HIV prevalence was 21% (196/926), HBsAg positivity 5% (47/936), HCV seroprevalence 55% (513/937), HCV viraemic prevalence (proportion tested with detectable HCV) 43% (404/937) and HCV viraemic rate (proportion HCV antibody positive with detectable HCV) 79% (404/513). HCV genotype 1a (73%, 270/368) was the most prevalent. In multivariate analysis, HCV infection was positively associated with residing in Pretoria (adjusted odds ratio (aOR) 1.27, 95% CI 1.21–1.34), living on the street (aOR 1.90, 95% CI 1.38–2.60), frequent injecting (aOR 1.58, 95% CI 1.15–2.16) and HIV infection (aOR 1.69, 95% CI 1.15–2.47), and negatively associated with black race (aOR 0.52, 95% CI 0.36–0.74) and sexual activity in the previous month (aOR 0.61, 95% CI 0.42–0.88).

**Conclusions:**

HCV and HIV are major health threats affecting PWID in these cities. Access to OST and needle and syringe services needs to be increased and integrated with HCV services. Social and structural factors affecting PWID who live on the street need to be addressed.

## Background

Globally, viral hepatitis is responsible for the deaths of approximately 1.34 million people every year, similar to the annual number of deaths from HIV/AIDS (1.3 million), malaria (0.9 million) and tuberculosis (1.3 million) [1–3]. In sub-Saharan Africa, approximately 60 million people are chronically infected with the hepatitis B virus (HBV) and 10.2 million people are chronically infected with hepatitis C virus (HCV) [1]. The region remains an epicentre for HIV with 25.7 million people living with HIV [2]. Most HBV infections occur in childhood through horizontal transmission, while most HIV infections occur during adolescence and adulthood through sexual contact [4–6]. Most HCV infections occur parenterally, through exposure to blood or unsafe medical practice [1, 5, 6]. People who inject drugs (PWID) who cannot access harm reduction services, specifically needle and syringe programmes and opioid substitution therapy (OST), are at particularly high risk of HCV, HIV and if non-immune, for HBV infection [7, 8]. In 1995, universal HBV vaccination was introduced in South Africa’s Expanded Programme of Immunisation [9]. Many current PWID in South Africa were born before this vaccine was introduced and hence a pool of chronic HBV remains, with HCV and HIV providing the bulk of new blood borne infections among PWID [9].

Screening for these infections includes the use of antibody and/or antigen tests, either point-of-care or laboratory-based. HIV confirmatory diagnosis in the sub-Saharan African context (a generalised HIV epidemic) usually involves a second, highly specific, rapid test from a different manufacturer [10]. A positive HBV surface antigen (HBsAg) is the marker for current infection, with further HBV serological assessment used to guide treatment. For HCV, confirmation of active viraemia is usually based on demonstrating HCV ribonucleic acid (RNA) on polymerase chain reaction (PCR) testing. Furthermore, HCV genotyping has until recently been performed to assess which of the six most frequent HCV genotypes is present to inform treatment choice. However, genotyping is not necessarily routinely required if pan-genotypic direct acting antivirals (DAAs) are available [8, 11].

Chronic HCV infection is associated with significant morbidity and mortality due to associated risks for cirrhosis and hepatocellular carcinoma [1]. Hepatitis C is readily treatable, and in most cases curable, with DAAs but access to therapy in South Africa is currently limited as these medications have yet to be registered, and a national viral hepatitis programme has yet to be fully implemented.

### Viral hepatitis and HIV among people who inject drugs

In sub-Saharan Africa, there were an estimated 1.4 million (95% uncertainty interval [UI] 0.6–3.8 million) PWID aged 15–64 years in 2017. Opioids (mostly heroin) are the most commonly injected drug in the region (78%), followed by stimulants, including cocaine and methamphetamine (51%) [7]. The frequency and potential risks of injecting vary by type and quality of the substance used, access to OST, individual patterns of use and the severity of the substance use disorder [12].

The estimated HBsAg prevalence among PWID in sub-Saharan Africa is 4% (95% UI 2–6%); HCV seroprevalence 22% (95% UI 18–27%) and HIV 18% (95% UI 11–25%) [7].

In South Africa, as in many settings globally, people who use drugs are criminalised and face high levels of stigma and discrimination [13]. Access to harm reduction services is limited and almost exclusively provided by non-profit organisations. The first needle and syringe service in South Africa was established in Cape Town in 2012, focusing on men who have sex with men (MSM) [14]. In 2015, this service transitioned to a different service provider and was accessible to a wider range of PWID. In the same year, needle and syringe services were established in Durban and Pretoria [15] and small OST demonstration projects have been operating in these cities since 2017 [16]. To date, harm reduction services have focused on HIV risk reduction, with very little focus on HCV [13]. There are no accurate empirical estimates of the number of PWID in the country. Existing estimates based on data modelled from a national household survey are between 67,000 and 75,000 [17, 18] and PWID populations in Cape Town, Durban and Pretoria are estimated at 1517, 1245 and 4514, respectively [19]. Programmatic evidence indicates that the number of people injecting drugs is increasing [20].

HIV prevalence among PWID participating in a South African five city study in 2013 was 14% [21]. Data around HBV and HCV epidemiology among PWID in South Africa has been limited to programmatic data and small, single site studies. A private, not-for-profit health facility in Pretoria that screened 271 PWID in 2013/14 identified an HCV seroprevalence of 24% [22]. More recently, 27% of MSM that use drugs who participated in a study in Cape Town (*n* = 41) were HCV antibody positive. Among them, 80% had injected heroin or methamphetamine in the previous 3 months [14].

When this study was being planned, the South African National Department of Health (NDOH) was in the process of developing national viral hepatitis policy. However, PWID-focused hepatitis services were not included, largely due to limited local data.

This study aimed to assess the prevalence of HCV, HBV and HIV and their co-infections among PWID and related risk factors for HCV infection in three South African cities. It was also intended to provide key baseline information to inform national hepatitis policy and programming.

## Methods

We planned to recruit 960 PWID (320 in Cape Town, Durban and Pretoria, respectively). This sample size was based on available resources. People who inject drugs were defined as people having injected a substance for non-therapeutic purposes, irrespective of the type of drug injected or the mode of injection, in the previous 12 months. All participants had to be 18 years or older. Data collection took place between August 2016 and October 2017.

Study activities were integrated into existing HIV prevention and sexual health services provided by non-profit organisations targeting PWID in these cities. MSM were recruited in a targeted sub-study at different sites looking into HBV, HCV and HIV prevalence and risks in that population. These results are not included here.

**Fig. 1:**
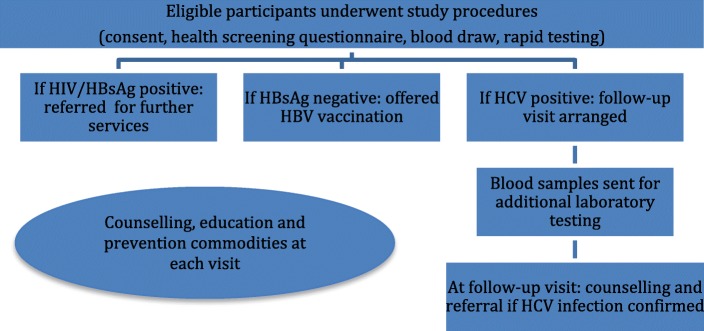
Summary of study procedures

An overview of study procedures is provided in Fig. 1. Participants who provided written informed consent were administered a confidential health screening questionnaire that was completed in English and clarified in the appropriate language as needed by the peers/nurses administering it. The questionnaire solicited demographic information (age, sex, race and housing), substance use (substances used in previous month and injecting in last month, including injecting frequency, needle and syringe reuse and sharing), use of OST and sexual risk behaviour.

Consenting participants had 20 ml whole blood drawn. HIV, HBV and HCV point-of-care (POC) testing was performed and samples from all HCV POC reactive individuals were sent to the National Institute for Communicable Diseases (NICD) in Johannesburg for confirmatory HCV enzyme-linked immunosorbent assay (ELISA), HCV PCR confirmatory viral load testing and genotyping. POC HIV testing was done in line with national protocols using a serial testing strategy [10]. POC HBsAg and HCV testing was performed using the Determine™ HBsAg (Alere Inc., MA, USA) and OraQuick® HCV rapid antibody test (Orasure Technologies Inc., PA, USA), respectively. All samples that were sent to the laboratory were subjected to anti-HCV testing (ARCHITECT Anti-HCV assay) on the ARCHITECT i1000SR system following the manufacturer’s instructions (Abbott Laboratories, Diagnostics Division, IL, USA). Samples that tested antibody positive were tested quantitatively using the COBAS® AmpliPrep/COBAS® TaqMan® HCV Quantitative Test v2.0 on the COBAS® Ampliprep Taqman® Analyser (Roche Molecular Systems, CA, USA). For HCV genotyping, 10 μl of biotinylated PCR amplicon was applied to nitrocellulose strips using the Versant HCV genotyping 2.0 assay (LiPA, Innogenetics, Ghent, Belgium). Genotyping results were interpreted using the reading card over the strip and the reading chart provided in the assay.

Participants received counselling around viral hepatitis and were provided with condoms, lubricant, information and education material and sterile injecting equipment. Participants diagnosed with HIV were referred to HIV care services. Those with a positive HBsAg screening test were referred to a pre-identified health facility for work-up and assessment for treatment. Participants with a negative HBsAg POC test were offered HBV vaccination. Those with a positive HCV screening test were given a follow-up date to return to obtain the results of additional laboratory testing. At the follow-up visit, the study nurse provided further counselling and linkage to care was arranged for additional work up and management of HCV infection. This was either at a designated gastrointestinal or liver clinic, where these existed, or at a pre-identified district hospital, where specialised clinics did not exist.

Data analysis was performed using STATA 11.2 (Statacorp LLC, Texas, USA). Data was initially analysed using descriptive statistics, stratified by city and biological sex. POC seroprevalence was calculated for HIV, HBV and HCV based on number of reactive POCs divided by the number of PWID participants that were tested as per protocol. HCV viraemic prevalence was calculated by dividing the number of participants with detectable viral load over the number of eligible PWID participants who had a HCV POC test. The HCV viraemic rate was calculated by dividing the number of participants with detectable viral load over the number of HCV POC positives. Bivariate analysis was done between HCV antibody status (reactive/ non-reactive) and selected demographic, drug using and sexual risk factors. Age and frequency of injecting were categorised into binary variables based on their median value. HCV antibody status was the outcome of interest in multivariate analysis. The model was adjusted for biological sex, age, race, city, living on the street, injecting practices (frequency, new needle at last injection, sharing needle at last injection), sexual practices (sexually active in last month, condom use at last penile-vaginal sex) and HIV status.

The study was approved by the Human Research Ethics Committee of the University of Cape Town (ref: 004/2016), the Research Ethics Committee of the University of the Witwatersrand (ref: M160510) and the Western Cape (ref: WC_2016RP19_818) and KwaZulu-Natal (ref: KZN_2016RP59_986) Provincial Department of Health Ethics Committees. Participants were not remunerated for their participation.

## Results

### Sociodemographics

Nine hundred and forty-three PWID were included in the per protocol analysis. The majority (87%, 819/943) were male, the median age was 29 and most were black (41%, 388/943) and living on the street (66%, 626/943). Demographic characteristics among PWID participants were similar across the three cities, apart from proportionately more people of mixed ancestry and fewer black participants in Cape Town than in the other sites, and a higher proportion of PWID living on the street in Durban (Table 1).

**Table 1:** Participants’ sociodemographic characteristics (*n* = 943)

	Cape Town	Durban	Pretoria	Total
N	301	318	324	943
Age [median (IQR)]	31 (28–35)	27 (25–31)	30 (26–34)	29 (26–34)
Sex [*n* (%)]				
Male	252 (84%)	287 (90%)	280 (86%)	819 (87%)
Female	49 (16%)	31 (10%)	44 (14%)	124 (13%)
Race [*n* (%)]				
Black	12 (4%)	169 (53%)	207 (64%)	388 (41%)
Mixed ancestry	215 (73%)	33 (10%)	11 (3%)	259 (28%)
White	68 (23%)	82 (26%)	103 (32%)	253 (27%)
Indian	1 (0%)	34 (11%)	1 (0%)	36 (4%)
Living on the street [*n* (%)]	163 (54%)	246 (77%)	217 (67%)	626 (66%)

### Substance use

The most frequently reported illegal substance used in the previous month was heroin (86%, 811/943). Methamphetamine or amphetamine-type stimulants (ATS) were the second most commonly used type of drug (28%, 262/943), with notably higher frequency of use in Cape Town compared to the other cities. Almost all (94%, 886/943) the participants had injected heroin in the previous month. Two-thirds (69%, 649/943) of all participants reported injecting any drug four or more times per day. At their last injection, most participants (77%, 722/943) reported using a new needle and syringe. A fifth (17%, 163/943) reported sharing needles at their last injection. Five percent (43/943) of participants had been on OST for at least 30 days at the time of the study (Table 2).

**Table 2:** Substance use practices among PWID (*n* = 943)

	Cape Town (*n* = 301)		Durban (*n* = 318)		Pretoria (*n* = 324)		Total (*n* = 943)	
	Male (*n* = 252)	Female (*n* = 49)	Male (*n* = 287)	Female (*n* = 31)	Male (*n* = 280)	Female (*n* = 44)	Male (*n* = 819)	Female (*n* = 124)
Substances used in last month								
Heroin	229 (91%)	47 (96%)	283 (99%)	31 (100%)	192 (69%)	29 (66%)	704 (86%)	107 (86%)
Methamphetamine /ATS	204 (81%)	39 (80%)	8 (3%)	0	10 (4%)	1 (2%)	222 (27%)	40 (32%)
Cannabis	29 (12%)	4 (8%)	19 (6%)	5 (16%)	70 (25%)	14 (32%)	118 (14%)	23 (19%)
Nyaope (heroin, cannabis mixture)	0	0	6 (2%)	2 (6%)	92 (33%)	14 (32%)	98 (12%)	16 (13%)
Cocaine	5 (2%)	1 (2%)	7 (2%)	2 (6%)	86 (32%)	13 (30%)	98 (12%)	16 (13%)
Methcathinone	1 (0%)	0 (0%)	0	0	7 (3%)	3 (7%)	8 (1%)	3 (2%)
Injected drugs in last month	239 (95%)	46 (93%)	282 (98%)	31 (100%)	269 (96%)	41 (93%)	790 (96%)	118 (95%)
Drug last injected								
Heroin	224 (89%)	44 (90%)	279 (97%)	31 (100%)	267 95%)	41 (93%)	770 (94%)	116 (93%)
Methamphetamine	15 (6%)	2 (4%)	2 (1%)	0	0	0	17 (2%)	2 (2%)
Average frequency of injecting (≥ 4 times per day)	193 (77%)	37 (76%)	174 (61%)	18 (58%)	204 (73%)	25 (60%)	569 (69%)	80 (66%)
New needle/injection last time	185 (73%)	34 (69%)	260 (91%)	27 (87%)	181 (65%)	35 (79%)	626 (76%)	96 (77%)
Shared needle last time	46 (18%)	18 (37%)	56 (20%)	3 (10%)	35 (13%)	5 (11%)	137 (17%)	26 (21%)
Currently on OST for ≥ 30 days	10 (4%)	1 (2%)	10 (3%)	2 (6%)	17 (6%)	3 (7%)	37 (5%)	6 (5%)

*ATS* amphetamine type stimulant

### Sexual risk practices

Forty-three percent (410/943) of the participants reported sexual activity in the previous month; higher among females (62%, 77/124) than males (41%, 333/819). Females and males reported similar numbers of sexual partners in the last week (median of 1, IQR 1–2 for females and median of 1, IQR 0–1 for males). Seven participants (four males, three females) reported receptive anal intercourse in the previous month. About half (52%, 212/410) of the participants reported condom use at last penile-vaginal sex, and 6% (24/410) reported exchanging drugs or goods for sex (13 males and 11 females). Thirty-eight percent of males (127/333) and 49% of females (38/77) reported alcohol or other substance use during their last sexual encounter, which was notably lower in Durban compared to the other cities (Table 3).

**Table 3:** Sexual risk practices among those sexually active in last month (*n* = 410)

	Cape Town (*n* = 144)		Durban (*n* = 178)		Pretoria (*n* = 88)		Total (n = 410)	
	Male (*n* = 109)	Female (*n* = 35)	Male (*n* = 157)	Female (*n* = 21)	Male (*n* = 67)	Female (*n* = 21)	Male (*n* = 333)	Female (*n* = 77)
Condom used at last penile-vaginal sex	59 (54%)	15 (43%)	84 (54%)	15 (71%)	28 (42%)	11 (52%)	171 (51%)	41 (53%)
Receptive anal intercourse in last week	2 (2%)	2 (6%)	0	0	2 (3%)	1 (5%)	4 (1%)	3 (4%)
Drugs/goods in exchange for sex in last month	9 (8%)	5 (14%)	1 (1%)	0	3 (4%)	6 (29%)	13 (4%)	11 (14%)
Alcohol or substance use at last sex	72 (66%)	21 (60%)	2 (2%)	0	53 (42%)	17 (81%)	127 (38%)	38 (49%)

### Testing results

Details of the testing results are provided in Table 4. A fifth of the participants (20%, 188/926) had their first HIV test as part of this study. HIV prevalence was 21% (196/926); 26% (31/119) among females and 20% (165/807) among males. HIV prevalence ranged from 7% (20/290) in Cape Town to 38% (122/319) in Pretoria. HIV prevalence was similar between male and female participants in Cape Town and Pretoria, with notably higher HIV prevalence among females than males in Durban (39%, 12/31 versus 15%, 42/286). HBsAg positivity was 5% (47/936); 5% (43/814) among males and 3% (4/122) among females, and similar across cities. Overall, anti-HCV positivity was 55% (513/937). HCV RNA was detected in 404 of the 513 participants who were anti-HCv positive yielding a viraemic rate of 79%. There was however marked variation in the HCV viraemic prevalence across cities, ranging from 35% in Durban, 44% in Cape Town to 84% in Pretoria. Overall, 12% (113/926) were HCV-HIV co-infected, 2% (21/936) HCV-HBV co-infected, 2% (14/925) HIV-HBV co-infected and eight participants were HCV-HBV-HIV triple-infected.

**Table 4:** HIV, HBsAg, anti-HCV, HCV RNA detected and HCV viraemic rate among PWID (*n* = 943)

	Cape Town (*n* = 301)		Durban (*n* = 318)		Pretoria (*n* = 324)		Total (*n* = 943)	
	Male (*n* = 252)	Female (*n* = 49)	Male (*n* = 287)	Female (*n* = 31)	Male (*n* = 280)	Female (*n* = 44)	Male (*n* = 819)	Female (*n* = 124)
HIV +ve	7% (16/244)	9% (4/46)	15% (42/286)	39% (12/31)	39% (107/277)	36% (15/42)	20% (165/807)	26% (31/119)
HBsAg + ve	7% (17/249)	0 (0/49)	4% (12/287)	3% (1/31)	5% (14/278)	7% (3/42)	5% (43/814)	3% (4/122)
Anti-HCV + ve POC	43% (108/ 250)	47% (23/49)	35% (101/287)	35% (11/31)	85% (237/278)	79% (33/42)	55% (446/815)	55% (67/122)
HCV RNA detected	34% (86/250)	27% (13/49)	28% (81/287)	26% (8/31)	71% (196/278)	48% (20/42)	46% (363/815)	34% (41/122)
HCV viraemic rate	80% (86/108)	57% (13/23)	80% (81/101)	73% (8/11)	83% (196/237)	61% (20/33)	81% (363/446)	61% (41/67)
HCV-HIV co-infection	1% (3/244)	2% (1/46)	6% (18/286)	13% (4/31)	28% (77/277)	24% (10/42)	12% (98/807)	13% (15/119)
HCV-HBV co-infection	3% (8/249)	0	2% (5/287)	0	2% (6/278)	5% (2/42)	2% (19/814)	2% (2/122)
HIV-HBV co-infection	1% (2/243)	2% (4/46)	2% (4/286)	3% (1/31)	2% (5/277)	5% (2/42)	2% (11/806)	3% (3/119)

HCV genotyping was possible on 96% (386/401) of the samples with detectable viral load. The most prevalent HCV genotypes were 1a (66%), 3a (14%), 3 (3%) and 1 (3%). Significantly, no genotype 5 was detected.

### Bivariate and multivariate analysis

The results from the bivariate and multivariate analysis are shown in Table 5. In bivariate analysis, positive HCV seroprevalence was positively associated with residing in Pretoria (OR 1.26, 95% CI 1.21–1.32, *p* < 0.001), living on the street (OR 1.59, 95% CI 1.21–2.09, *p* = 0.001) and injecting four or more times per day (OR 1.67, 95% CI 1.25–2.19, *p* < 0.001). HCV infection was negatively associated with the use of new injecting equipment at last injection (OR 0.70, 95% CI 0.51–0.94, *p* = 0.020), sexual activity in the last month (OR 0.40, 95% CI 0.31–0.52, *p* < 0.001) and condom use at last penile-vaginal sex (OR 0.48, 95% CI 0.35–0.65, *p* < 0.001).

**Table 5:** Associations with HCV serostatus among PWID in three South African cities (*n* = 943)

	OR	95% CI	*P* value	aOR	95% CI	*P* value
Age (≥ 29 years)	1.21	0.93–1.56	0.151	1.00	0.74–1.36	0.995
Male sex	1.03	0.70–1.50	0.889	1.01	0.65–1.56	0.966
Black race	1.13	0.87–1.46	0.373	0.52	0.36–0.74	< 0.001
Pretoria	1.26	1.21–1.32	< 0.001	1.27	1.21–1.34	< 0.001
Living on the street	1.59	1.21–2.09	0.001	1.90	1.38–2.60	< 0.001
Injects ≥ 4 times per day	1.67	1.25–2.19	< 0.001	1.58	1.15–2.16	0.005
Used new needle at last injection	0.70	0.51–0.94	0.020	0.87	10.61–1.23	0.416
Shared needle at last injection	1.06	0.96–1.17	0.254	0.96	0.86–1.07	0.414
Sexually active in last month	0.40	0.31–0.52	< 0.001	0.61	0.42–0.88	0.008
Condom at last penile-vaginal sex	0.48	0.35–0.65	< 0.001	0.85	0.57–1.28	0.432
HIV infection	1.08	0.78–1.49	0.636	1.69	1.15–2.47	0.008

The frequency of injecting among men was significantly lower among those who were sexually active compared to men who had not been sexually active in the previous month (OR 0.58, 95% CI 0.43–0.79, *p* < 0.001). Furthermore, men who were sexually active were more likely to have used a new needle and syringe the last time they injected compared to men who were not sexually active (OR 1.47, 95% CI 1.05–2.06, *p* = 0.024). Drug using risk practices among women were similar to those among men when analysed in relation to their sexual activity in the last month.

In multivariate analysis, positive HCV serostatus was positively associated with residing in Pretoria (aOR 1.27, 95% CI 1.21–1.34, *p* < 0.001), living on the street (aOR 1.90, 95% CI 1.38–2.60, *p* < 0.001), frequent injecting (aOR 1.58, 95% CI 1.15–2.16, *p* = 0.005) and being HIV infected (aOR 1.69, 95% CI 1.15–2.47, *p* = 0.008), and negatively associated with black race (aOR 0.52, 95% CI 0.36–0.74, *p* < 0.001) and being sexually active in the previous month (aOR 0.61, 95% CI 0.42–0.88, *p* = 0.008).

## Discussion

This is the largest published quantitative study of HBV, HCV and HIV among PWID in South Africa to date. The study demonstrates a significantly higher prevalence of HCV infection among PWID (43%) than previously known [23]. Notably, HCV prevalence among PWID in Pretoria was substantially higher than in other cities. This study did not explore the reasons for this geographical variation. However, expert consultation in other settings has suggested that injecting practices as a drug using culture have been in existence for longer in Pretoria than other cities, potentially suggesting a reason for this finding [24].

A large proportion of the participants lived on the street, accounted for by the recruitment of participants through existing harm reduction services that target people from lower socio-economic circumstances. People who live on the street often have limited financial resources to purchase injecting equipment, contributing to the increased likelihood of reuse, sharing and use of contaminated injecting equipment [25]. People living on South African streets who use drugs have little or no access to private spaces to do so, and frequently experience human rights violations, including assault, confiscation of possessions (including injecting equipment) and being moved [26–28]. Consequently, conditions for safe injecting are limited. The multivariate analysis found an increased likelihood of HCV infection among people living on the street, reinforcing the importance of addressing social and structural factors to enhance HIV and HCV health outcomes.

The study found that HCV risk is positively associated with increased injection frequency. This is supported by the per injection risk without 100% access to sterile injecting equipment [29]. The high level of reported use of new needles is surprising, and may reflect a social desirability bias, considering that the majority of people inject four or more times a day and access to sterile injecting equipment is limited. Existing harm reduction services in these cities distribute between 10 and 14 sterile needles and syringes at each encounter with a PWID client, with these projects aiming to visit locations where PWID are provided with services once or twice a week.^1^ An assessment of needle and syringe service coverage in Pretoria and Cape Town was completed in 2017 and results are forthcoming [24]. The reported levels of needle and syringe sharing highlight the need for increased saturation of needle and syringe services.

Overall, few PWID (5% of the sample) had access to OST in these cities, which is to be expected as OST remains in the pilot stages [16]. It is also unclear if the reported OST access was sustained or over limited periods and if the dosage was aligned to global recommendations [8].

High coverage of OST and needle and syringe services can reduce the risk of HCV infection by 74% [30]. Modelling data from Kenya (PWID population of 50,000 with HCV prevalence of 11%) shows that providing 75% needle and syringe coverage and 50% OST would reduce the risk of HCV incidence among PWID by 69% by 2030, and elimination targets would be reached through the addition of treating chronic HCV infections among 1000 PWID over 5 years [31].

This research confirmed the findings of a previous study that documented elevated HIV prevalence among PWID compared to the general population [21]. In multivariate analysis, HIV infection is significantly associated with HCV serostatus, highlighting the shared transmission routes of these viruses and the need to integrate viral hepatitis and HIV services for PWID. The study also documents HBsAg prevalence similar to the general population [4]. While a relatively small proportion of people were found to be co-infected with HBV/HCV/HIV, the added morbidity and mortality requires that co-infection remains an important area for consideration. The relative exclusive prevalence of HCV genotypes 1a and 3a is similar to that in other countries where HCV infection is predominantly spread through sharing contaminated injecting equipment among PWID [32, 33]. However, of note in South Africa, genotype 5a (a predominant genotype in patients with liver disease [34]) was not found among PWID and suggests that 5a circulates in the general population and not in PWID.

Participants in this study were recruited from among those who currently access or who were reachable to organisations implementing harm reduction services, specifically mobile needle and syringe distribution and collection services in these cities. Unsurprisingly, the participants were overwhelmingly male given that globally, a higher proportion of PWID are male compared to female, with the proportion of female PWID ranging from 3% in South Asia to 33% in Australasia, and 12% in sub-Saharan Africa [7]. Programmatic data from the services that operate in these cities reflects that between 10 and 13% of service users are women [35]. While this is possibly due to higher numbers of men who inject drugs than women in South Africa, it also likely reflects barriers preventing women who inject drugs from accessing harm reduction services—including stigma, discrimination and services that do not address their specific needs [36]. Previous studies among South African and African PWID also included a smaller proportion of women who inject drugs [21, 37]; however, globally, a higher proportion of women who inject drugs are reached [38]. Nevertheless, there is global [39] and sub-Saharan [40] recognition of the need for gender-appropriate and specific HIV and HCV services for women who inject drugs due to their specific needs.

A diverse cross-section of South African racial groups was recruited across sites, indicating that substance use and injecting practices are issues affecting people across race or ethnicity. The relative order of the size of racial groups in the study sites reflected the demographic characteristics of the relevant cities [41]. However, the relative over-representation of white PWID remains (27% of the sample, versus 8% of the general population) [41]. It is not clear from this study if there are relatively more white PWID or if this is due to selection bias. Most of the study sites where centrally located in areas where PWID congregate. Non-white PWID living in poorer, peripheral areas of the city may have not been reached by existing harm reduction services and therefore not recruited in the study. There is no published evidence supporting different levels of stigma around injecting drug use among different racial groups in these cities, but this may also have been a factor. It is also unclear why lower HCV risk was found among black PWID. Data was not collected on length of participant injecting history, so no analysis can be made on associations between injecting history and race groups as a proxy for other factors.

The findings from this study around substance use reflect substance use treatment data that shows the prevalence of poly drug use in South Africa and different usage patterns across the country [35]. Study and treatment data point to heroin being the most commonly injected drug, and methamphetamine/ATS injecting being highest in Cape Town.

Proportionately more women than men reported sexual activity in the previous month and condom use was similarly sub-optimal. More women than men reported transactional sex for drugs, which has previously been identified among women who inject in South Africa [21].

Interestingly, men who reported recent sexual activity showed lower risk drug use practices than their counterparts who did not report being sexually active. This may be because higher frequency of opioid use is associated with reduced libido, and can induce impotence among men [42]. It may therefore be that men engaging in regular sexual activity are those using less opioids, and are therefore able to exercise greater care with each instance of injection. It may also be that sexual activity is indicative of the existence of intimate partnerships that impact on risky injecting behaviours. While intimate couples who inject drugs together often engage in needle sharing and higher risk injecting practices within the couple [43], there is also increasing evidence that drug use tends to lessen when people have close social relationships and that couples set negotiated standards and limits to such use [44]. This can include agreements about where, when, how, and with whom drugs are injected [45]. Furthermore, concerns for placing primary sexual partners at risk of infectious diseases may contribute to safer injecting [13]. These limits on injecting behaviour in intimate relationships may be some of the reasons that recent sexual activity was found to be generally protective against HCV infection.

### Limitations

The opportunistic nature of the sampling method prevents extrapolation to larger PWID populations in these cities and beyond. However, the findings confirm previous local studies and global experience. Information bias may have influenced the reliability of measures assessing substance use and sexual activity (including under reporting of anal sex and exchange of sex for drugs and/or goods). The low frequency of reported alcohol or substance use at the time of sex in Durban is suspected to be an underestimation, as other sexual risk practices were similar to the other cities. This difference could be attributable to several factors. One factor may have been due to differential solicitation of this question by study team members in Durban compared to the other cities. Another potential explanation is potential differences in social norms around discussing sexual practices among PWID in the various cities; however, there is no published evidence supporting this. The researchers were experienced in working with PWID so PWID were likely to feel safe in participation; however, the reporting of favourable (less risky) practices may have been encouraged by the fact that participants were engaging with the same people who provided harm reduction services. This may have resulted in underestimation of HCV risks and over-estimation of access to and use of needle and syringe services.

## Conclusions

HCV, and to a lesser extent HIV, are major health threats affecting PWID in South Africa. On-going high risk practices are influenced by limited access to OST and needle and syringe services, as well as the social and structural factors affecting PWID who live on the street in the context of criminalisation of people who use drugs. Furthermore, in the absence of HCV treatment, an increase in HCV infections is inevitable without a rapid, targeted response that is appropriate and acceptable to PWID. Programmatic responses should also focus on prioritising treatment within Pretoria where HCV and HIV prevalence is highest, with intensified prevention messaging in that city. However, hepatitis awareness and prevention activities need to be up-scaled in other cities where HCV prevalence has not yet reached such high proportions.

Intensified efforts are needed to reach and provide services to women who inject drugs and to ensure that these services are inclusive of their sexual and reproductive health needs. Tailored outreach and viral hepatitis testing and treatment services, facilitated by gender appropriate teams should be considered as services scale up.

Broader social issues related to living conditions and social isolation need to be considered in holistic responses to HCV and HIV infection among PWID in these cities. Interventions that address structural issues that may be contributing to the elevated HCV risk among PWID living on the street are needed, which are not limited to exploration of safe injecting facilities, and enhanced support from law enforcement for needle and syringe services.

This study was limited to a description of HCV, HBV and HIV among PWID in these cities that have (potential) access to harm reduction services. Additional research is needed on the epidemiology of these infections among PWID in other South African contexts (urban and rural), as well as within the correctional services system. Greater insights are also needed into factors associated with on-going parenteral risk in the presence of harm reduction services. Models that explore innovative ways to provide integrated HCV-HBV-HIV and other holistic services to PWID in the South African context are needed to inform the granular detail and implementation experience that will be necessary for an effective HCV response. Future research is needed to understand health care providers’ knowledge around viral hepatitis and HIV among PWID.

Eliminating viral hepatitis as a public health threat in South Africa by 2030 will only be an attainable reality once hepatitis prevention, testing and treatment services are provided to all PWID as needed. Services will need to include access to sterile injecting equipment, OST and HIV and HCV testing and treatment. Like many other counties, South Africa has the guidelines and a national action plan to address this issue, but political support, appropriate resource allocation and dedicated and passionate people that include the PWID community will be required to support implementation and reach this ambitious goal.

## Abbreviations

AIDS: Acquired immunodeficiency syndrome
aOR: Adjusted odds ration
ATS: Amphetamine-type stimulants
CI: Confidence interval
CPT: Cape Town
DAA: Direct acting antiviral
DBN: Durban
ELISA: Enzyme-linked immunosorbent assay
HBsAg: HBV surface antigen
HBV: Hepatitis B virus
HCV: Hepatitis C virus
HIV: Human immunodeficiency virus
MSM: Men who have sex with men
NDOH: South African National Department of Health
NICD: National Institute for Communicable Diseases
OST: Opioid substitution therapy
PCR: Polymerase chain reaction
POC: Point-of-care
PTA: Pretoria
PWID: People who inject drugs
RNA: Ribonucleic acid
UI: Uncertainty interval
WHO: World Health Organisation

## Footnotes

1 Personal communication with Andrea Schneider, Step Up Project national coordinator (4 December 2018).
